# Chito-Oligosaccharide Inhibits the De-Methylation of a ‘CpG’ Island within the Leptin (*LEP*) Promoter during Adipogenesis of 3T3-L1 Cells

**DOI:** 10.1371/journal.pone.0060011

**Published:** 2013-03-27

**Authors:** Bojlul Bahar, John V. O’Doherty, Alan M. O’Doherty, Torres Sweeney

**Affiliations:** 1 School of Agriculture and Food Science, University College Dublin, Dublin, Ireland; 2 School of Veterinary Medicine, University College Dublin, Dublin, Ireland; Virginia Tech, United States of America

## Abstract

Chito-oligosaccharide (COS) is a natural bioactive compound, which has been shown to suppress lipid metabolic genes and lipid accumulation in differentiating adipocytes. Leptin has been identified as a key regulator of energy homeostasis and is known to be under epigenetic regulation during adipogenesis. Hence, the first objective of this experiment was to compare leptin gene (*LEP*) expression and leptin secretion during the different stages of adipogenesis and to investigate the effect of COS on these processes. As COS inhibited *LEP* expression during adipogenesis, the second aim was to investigate the methylation dynamics of a ‘CpG’ island in the proximal region of the *LEP* promoter during adipogenesis and to determine the effect of COS on this process. Mouse 3T3-L1 cells were stimulated to differentiate in the absence or presence of COS and the levels of leptin mRNA and protein were evaluated on days 0, 2, 4 and 6 post-induction of differentiation (PID). The extent of de-methylation of six CpG sites was evaluated. *LEP* mRNA transcript and protein could not be detected on either day 0PID or 2PID. In contrast, both were detected on day 4PID (*P*<0.05) and 6PID (*P*<0.001) and both were inhibited by COS (P<0.001). Of the six CpG sites analyzed, CpG_52, CpG_62 and CpG_95 became 11.5, 5.0 and 5.0% de-methylated between day 2PID and 6PID, respectively. COS blocked this de-methylation event at CpG_52 (*P*<0.001), CpG_62 (*P*<0.01) and CpG_95 (*P*<0.01) on day 6PID. These data suggest that COS can have an epigenetic effect on differentiating adipocytes, a novel biological function of COS which has potential applications for the manipulation of leptin gene expression, adipogenesis, and conditions within the metabolic syndrome spectrum.

## Introduction

Obesity is rapidly becoming an epidemic in both developed and developing nations of the world [1], [2]. It is characterized by a disruption in energy homeostasis, leading to the excessive accumulation of fat in adipocytes [3]. Excessive body fat is associated with a spectrum of diseases in the metabolic syndrome spectrum [4] including obesity and type-2-diabetes. The molecular mechanisms underlying the maturation of adipocytes involve the activation of biochemical pathways mediated through signaling molecules including cytokines, chemokines and adipokines [5]. Mouse 3T3-L1 cells are a widely used model of adipocytes [6] and are frequently used to elucidate various underlying cellular and molecular events involved in the process of adipogenesis [7], [8]. This cell line was previously used to identify the anti-adipogenic potential of a range of compounds including herbal drugs [9], [10] and neutraceuticals [11], [12], [13].

The *LEP* gene is switched on by epigenetic modulation when pre-adipocytes are stimulated to begin the process of adipogenesis [14], [15], [16]. The proximal promoter region of the *LEP* gene has a number of conserved methylation sites (CpG) which remain highly methylated in the pre-adipocyte and thereby prevent expression of the *LEP* gene at this stage of development [16], [17]. However, de-methylation of the *LEP* promoter occurs during the differentiation process, resulting in a loss of methyl groups from CpG sites of the *LEP* promoter in mature adipocytes [17]. This epigenetic modulation of the *LEP* promoter is a vital mechanism underlying absence of any leptin secretion in pre-adipocytes while driving abundant synthesis and secretion of leptin by mature adipocytes [18].

Differentiation of pre-adipocytes into mature adipocytes can be inhibited *in-vitro* following exposure to chito-oligosaccharide (COS) [11], [19]. COS is a polymer of glucosamine with a number of bioactive properties: recent studies have suggested that COS inhibits adipogenesis through altered expression of a number of key regulators of lipid metabolism, including leptin [11], [20]. However, this seems to be cell cycle stage dependent, as COS inhibits the expression of the *LEP* gene in the differentiating adipocyte [11], while glucosamine, a constituent of COS, can stimulate *LEP* gene expression in mature adipocytes [21]. Recently, we have demonstrated in the porcine model that dietary inclusion of chitosan, a parent compound of COS, suppressed body weight gain which was associated with elevated serum leptin concentrations [22]. Based on the facts that COS can inhibit the differentiation of pre-adipocyte to mature adipocyte, and that de-methylation of the *LEP* gene promoter is necessary for secretion of leptin by mature adipocytes, we hypothesized that COS interferes with the epigenetic modulation of the *LEP* gene promoter during adipocyte differentiation. Therefore, the first objective of this experiment was to compare *LEP* gene expression and leptin secretion during the different stages of adipogenesis and to investigate the effect of COS on these processes. The second aim was to investigate the methylation dynamics of a ‘CpG’ island in the proximal region of the *LEP* promoter during adipogenesis and to determine the effect of COS on this process.

## Materials and Methods

### Chito-oligosaccharide (COS)

Low molecular weight COS (5–10 kDa, >70% chitooligosaccharide content, >70% deacetylation) was purchased from Kitto Life Co. Ltd (Kyungki-do, Seoul, Korea). This source of COS inhibited the differentiation of mouse 3T3-L1 pre-adipocytes into mature adipocytes [19].

### Cell Culture

Mouse 3T3-L1 pre-adipocytes were obtained from the American Type Culture Collection (ATCC, Manassas, VA, USA). Cells were cultured in a Dulbecco modified eagle’s medium (DMEM, Gibco, Invitrogen Corp., San Diego, CA, USA) containing 10% fetal calf serum (Gibco) and 1% penistrep solution (Sigma-Aldrich Corp., St. Louis, MO, USA) in a 37°C humidified incubator with 5% CO_2_. During the multiplication phase of pre-adipocytes, the media was changed every alternate day and cells were trypsinized before reaching full confluence and plated afresh.

Pre-adipocytes were differentiated as described previously [19]. Supernatant and cells were collected relative to post induction of differentiation (PID). Pre-adipocytes were collected on day 0PID. Differentiating adipocytes were collected on days 2, 4 and 6PID.

COS was added to the media on day 0, 2, and 4 PID at final concentrations of 0, 600, 1200, 2400 and 4800 µg/ml.

### Measurement of Leptin

Leptin was measured in the supernatant on day 0, 2, 4 and 6PID in absence or presence of COS. Leptin was quantified using a mouse leptin sandwich ELISA (R&D Systems Europe, Ltd. Abingdon, UK) according to the manufacturer’s instructions. Signal detection was performed in a microtiter plate reader at an absorbance of 450 nm against 570 nm. Each measurement was performed in triplicate on three independent occasions.

### RNA Extraction

Pre- and differentiating adipocytes were harvested in TRI reagent (Applied Biosystems, Foster City, CA, USA) and total RNA was extracted using the Trizol method according to the manufacturer’s instructions. RNA was dissolved in 20 µl of nuclease-free water and then subjected to Deoxyribonuclease I (Sigma-Aldrich) treatment to eliminate the genomic DNA contamination. Column purification of RNA was performed using the GenElute mammalian total RNA miniprep kit (Sigma-Aldrich). The quality and quantity of total RNA were assesed by analyzing 1 µl of total RNA on an Agilent 2100 Bioanalyser (Agilent Technologies, Inc., Santa Clara, CA, USA) using RNA Nano LabChips (Caliper Technologies Corporation, Hopkinton, MA, USA). All RNA samples used for the gene expression study had an RNA integrity value ≥8·0.

### Quantitative Real-time PCR

cDNA synthesis was performed with 1 µg of total RNA, using the RevertAid H minus first-strand cDNA synthesis kit (Fermentas GmbH, St Leon-Rot, Germany) following the manufacturer’s protocol. Quantitative expressions of *LEP* gene as the target and glyceraldehyde 3-phosphate dehydrogenase (*GAPDH*) and β-actin (*ACTB*) as reference genes were evaluated as described previously [19].

### Bisulfite Modification of DNA and Pyrosequencing

Based on observations that COS can inhibit the differentiation of pre-adipocytes to mature adipocytes, and de-methylation of the *LEP* promoter is necessary for production of leptin by mature adipocytes, we investigated the methylation pattern at the CpG sites present in a 134 bp region of the mouse *LEP* promoter. For this, the pre-adipocyte was allowed to differentiate as described previously [19] and differentiating pre-adipocytes were treated with 0, 600, 1200, 2400 and 4800 mg/ml COS and harvested on day 2, 4 and 6PID. Genomic DNA was extracted using the Wizard Genomic DNA Purification Kit (Promega Corp. Madison, WI, USA) following manufacturer’s instructions.

Genomic DNA was bisulfite modified, according to the manufacturer’s guidelines, using the EZ DNA Methylation-Direct™ Kit (Zymo Research, Irvine, CA, USA). About 200 ng of DNA (in a 20 µl volume) was modified directly by mixing the sample with 130 µl CT reagent (sodium bisulfite conversion solution) and incubating at 98°C for 8 min, followed by 64°C for 8 hr. Modified DNA samples were transferred directly to Zymo-Spin IC columns pre-loaded with 600 µl M-Binding Buffer and mixed by gentle pipetting. Columns were centrifuged at 12,000 rpm for 45 sec, the flow-through was discarded and the column was washed with 100 µl M-Wash Buffer. M-desulphonation buffer (200 µl) was added to the column and left to stand at room temperature for 30 min. Two final wash steps with 200 µl wash buffer were carried out before eluting the DNA in 40 µl M-Elution buffer.

Murine *LEP* pyrosequencing primers were designed using Pyromark assay design software (Qiagen) in accordance to the genomic DNA sequence of a 216 bp CpG island located upstream of the transcription start site, obtained using the UCSC genome browser (http://genome.ucsc.edu/). PCR primers were supplied HPLC purified by MWG Eurofins, the forward primer was 5′-biotinylated. A 134 bp amplicon, containing six CpG dinucleotides, was evaluated for methylation analysis.

Bisulfite PCR was carried out in 25 µl reactions containing 1 mM MgCl_2_, 0.2 µm each of the forward and reverse primers, 0.2 mM dNTPs, 1X PCR Buffer (minus Mg), Platinum *Taq* DNA polymerase and 3 µl bisulfite modified DNA. Cycling conditions were as follows: 95°C for 5 mins followed by 40 cycles of 95°C, 30 sec; 54°C for 30 sec; 72°C, 30 sec and a final elongation step of 5 min at 72°C. PCR products were verified by electrophoresis on a 2% (w/v) agarose gel. Verified PCR products (20 µl) were bound to 2 µl streptavidin coated sepharose beads (GE Healthcare, Waukesha, WI, USA) in a reaction containing 40 µl Binding buffer (Qiagen) and 20 µl nuclease free H_2_O (Promega). Pyrosequencing was performed as outlined before [23]. Complete bisulfite conversion of DNA samples was verified using an internal control at a ‘non-CpG’ cytosine during pyrosequencing.

### LEP Gene Promoter Assay

#### Promoter construct

A 1 kb region (−991 nt to +8 nt relative to the transcription start site) of the human *LEP* promoter containing the proximal promoter region was amplified by PCR. The forward primer (5′ CCTTAGATCTACCAGAATAGGCCTGGGTTC 3′) was designed to include a *Bgl II* restriction digest site (underlined). The reverse primer (5′ TAGTAAGCTTATTCCTACGGGGCTCCATGC 3′) incorporated a *Hind III* restriction digest site (underlined). Both forward and reverse primers have a four nucleotide anchor at the 5′ end. PCR amplification was performed in a PTC-225 DNA Engine Tetrad (MJ Research, Inc. Massachusetts, USA) using 1× Platinum PCR SuperMix High Fidelity (Invitrogen Corp., San Diego, CA, USA), 0.2 µM of each of the forward and reverse primers and 50 ng of human genomic DNA (Promega Corp.) in a final volume of 100 µL. The PCR cycle condition included an initial denaturation step of 94°C for 2 min followed by 39 cycles of 94°C for 45 sec, 60°C for 45 sec and 70°C for 1 min and a final extension of 72°C for 10 min. The PCR product (999 bp) was assessed on a 1.2% (w/v) agarose gel stained with ethidium bromide. The PCR product was cloned into firefly luciferase expression vector pGL4.17 (Promega Corp.) as described previously [19]. Transfection grade endotoxin free plasmid construct containing the human *LEP* promoter insert was prepared using EndoFree Plasmid Maxi Kit (Qiagen, Chatsworth, CA, USA).

#### Transfection assay

The day before the transfection assay, pre-adipocytes (6×10^5^ cells/ml) were initially cultured in DMEM containing 10% fetal calf serum in a 24 well cell culture plate. The transfection cocktail (for each well) contained 25 µl DMEM basal media, 0.3 µl FuGENE HD transfection reagent (Roche Diagnostics GmbH, Mannheim, Germany) and 100 ng of *LEP* promoter construct DNA. Following incubation at room temperature for 15 min, this cocktail was introduced drop wise onto the cells. Cells were grown on a DMEM containing 10% fetal calf serum for 48 hrs. Transiently transfected cells were treated with COS in a serum and antibiotic free basal medium.

Transiently transfected cells were allowed to grow for 24 hrs before harvest and luciferase assay was performed using the Luciferase Reporter Assay system (Promega Corp.). In brief, the media was removed and the cells washed with 500 µl of phosphate buffer saline. Lysis of cells was performed by adding 250 µl of passive lysis buffer (Promega Corp.) followed by incubation at 37°C in a shaking incubator for 30 min at 700 rpm. The firefly relative luciferase unit activity was measured in 20 µl of the cell lysate in a 20/20 n Single Tube Luminometer (Turner Biosystems, Inc. Sunnyvale, CA, USA).

*In-vitro* methylation of *LEP* gene: *In-vitro* methylation of the *LEP* promoter construct was performed using CpG Methyltransferase (M.SssI) enzyme (New England Biolabs Inc. Herts, United Kingdom). The methylation reaction contained 3 µg of the promoter construct DNA, 12U of SssI methylase and 1X NE Buffer 2 supplemented with 640 µM S-adenosylmethionine (SAM) in a final volume of 20 µl. The content was incubated at 37°C for 2 hr followed by stopping of the reaction by treating at 65°C for 20 min. The promoter construct was cleaned using Gen Elute PCR clean-up Kit (Sigma-Aldrich Corp.) following the manufacturer’s instructions.

### Statistical Analysis

Data for all the variables were checked for a normal distribution. Data on leptin protein abundance, *LEP* gene expression (fold change), % methylation were analysed using one-way ANOVA and means were compared by Tukey’s test. The promoter assay data were compared by student- t test.

## Results

### Quantification of LEP Gene Expression and Leptin Protein Secretion during Adipogenesis

**Figure 1:**
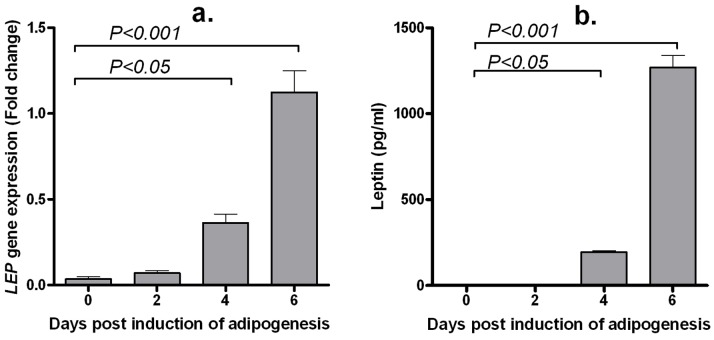
Abundance of mouse *LEP* mRNA transcripts (A) and protein (B) during adipogenesis. Mouse 33-L1 pre-adipocytes were induced to differentiate and cells were harvested for RNA isolation and quantitative expression of *LEP* gene on days 0, 2, 4 and 6 post-induction of differentiation. Cell culture media was harvested for quantification of leptin production on days 2, 4 and 6 post-induction of differentiation. Day 0 and day 6 correspond to pre-adipocyte and mature adipocytes, respectively. Day 2 and day 4 correspond to a phase where the cells were actively involved in the differentiating process. Data represents mean ± standard error from three independent replicate experiments.

LEP gene expression: The expression of the *LEP* gene was at the minimum limit of detection in pre-adipocytes (day 0PID) and early differentiation stage adipocytes (day 2PID) (Figure 1A). *LEP* mRNA transcript was detected on day 4PID (*P*<0.05), with a significant increase in gene expression evident on day 6PID (*P*<0.001).

Leptin protein secretion: Leptin was not detected in the supernatant on days 0PID or 2PID; Figure 1B). As adipocyte differentiation progressed, an increase in leptin secretion was evident: with leptin concentrations increasing from 193.7±14.35 pg/ml on day 4PID (*P*<0.05) to 1269.0±122.3 pg/ml on day 6PID (*P*<0.001) (Figure 1B).

### Effect of COS on Leptin Protein Secretion and LEP Gene Expression during Adipogenesis

**Figure 2:**
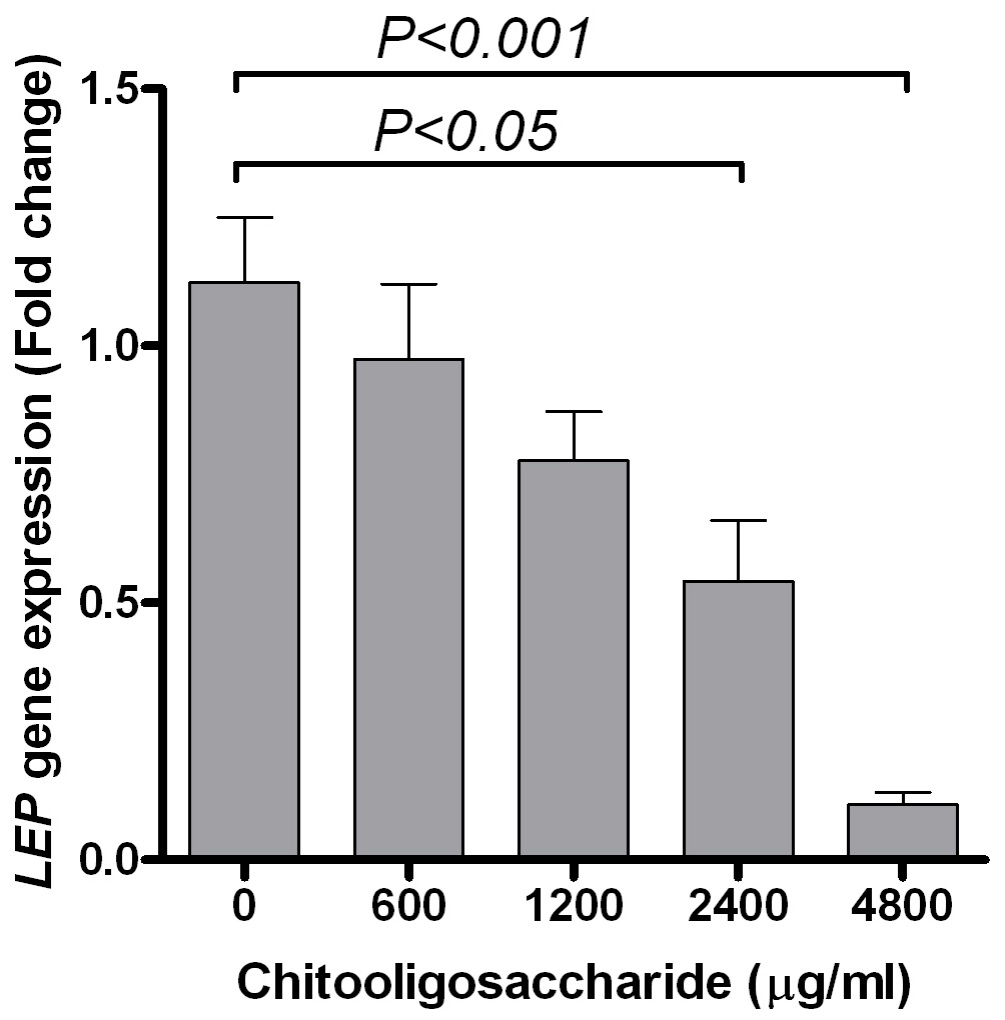
Effect of chitooligosaccharide on mouse *LEP* gene expression at day 6 post-induction of differentiation. Mouse 33-L1 pre-adipocytes were induced to differentiate in absence or presence of chitooligosaccharide and cells were harvested for RNA isolation and quantitative expression of *LEP* gene on day 6 post-induction of differentiation. Data represents mean ± standard error from three independent replicate experiments.

LEP gene expression: As day 6PID was determined to be the day of maximum leptin secretion in the control sample, the effect of COS on *LEP* gene expression was evaluated on day 6PID. The relative abundance of the *LEP* mRNA transcript was 1.12±0.13 fold in the control (COS 0 µg/ml) sample (Figure 2). A significant decrease in the abundance of the *LEP* mRNA transcript was evident with exposure to 2400 (0.54±0.12 fold, *P*<0.05) and 4800 (0.11±0.02 fold, *P*<0.001) µg/ml COS.

**Figure 3:**
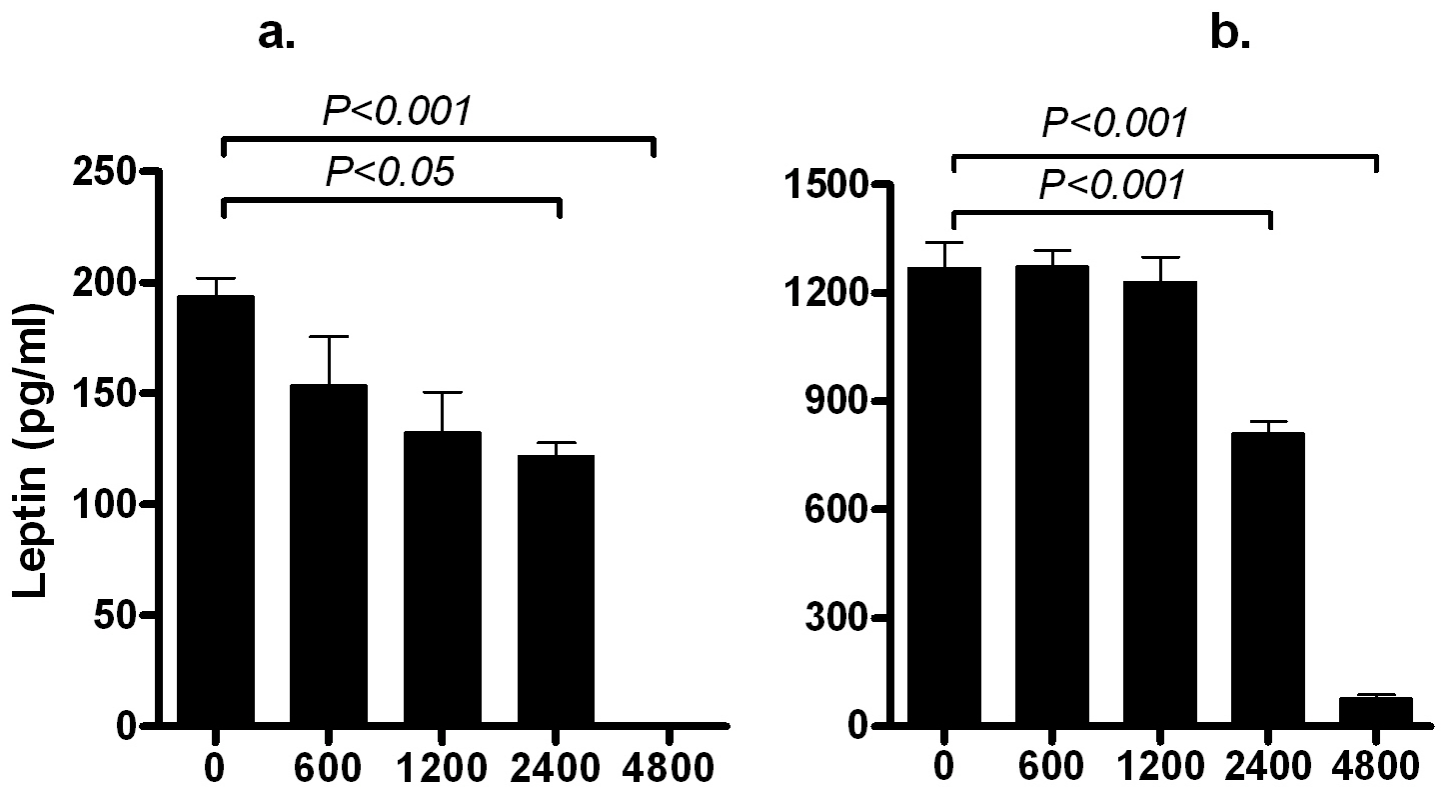
Effect of chitooligosaccharide (COS) on the production of leptin by 3T3-L1 pre-adipocytes during adipogenesis. Mouse 33-L1 pre-adipocytes were induced to differentiate in the absence or presence of chitooligosaccharide and cell culture media was harvested for quantification of leptin production on days 2–4 post-induction of differentiation (A) and days 4–6 post-induction of differentiation (B) Data represents mean ± standard error from three independent replicate experiments. Note the differences in the scale of Y-axis representing the leptin concentration (pg/ml) in the media for different figures.

Leptin protein secretion: As leptin was not detectable on day 0PID or 2PID, the effect of COS was not evaluated at these time points. COS inhibited leptin secretion on day 4PID and 6PID (*P*<0.001; Figure 3A and B). On day 6PID, when leptin concentrations were at its maximum in the control sample (COS 0 µg/ml: 1269.0±122.3 pg/ml), exposure to 600, 1200, 2400 and 4800 µg/ml of COS resulted in 1273.0±77.54, 1230.0±121.5, 810.9±59.33 (*P*<0.001) and 77.25±19.28 (*P*<0.001) pg/ml of leptin, respectively.

### De-methylation of the LEP Promoter during Adipogenesis

To determine the methylation status of the *LEP* gene promoter during adipogenesis, the extent of methylation of a ‘CpG’ island was evaluated on days 2, 4 and 6PID. Of the six CpG sites analyzed, de-methylation was observed at four CpG sites (CpG_52, CpG_62, CpG_81 and CpG_95) during differentiation. The most conspicuous CpG site de-methylated during the differentiation process was CpG_52, where 11.5% de-methylation was evident between day 2 and day 6PID. During this period, de-methylation was also observed at CpG_62 (5%), CpG_95 (5%) and CpG_81 (3%). No alternation in methylation status was observed at two CpG sites (CpG_1 and CpG_85).

### Effect of COS on the De-methylation of the LEP Promoter during Adipogenesis

**Figure 4:**
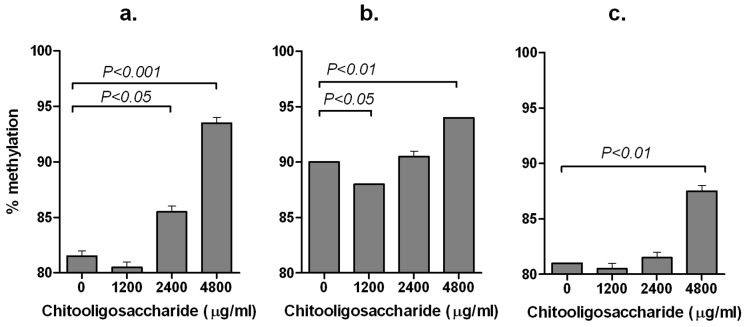
Effect of chitooligosaccharide on the de-methylation three CpG sites in the *LEP* promoter. Extent of methylation at CpG_52 (A), CpG_62 (B) and CpG_95 (C) of the mouse *LEP* promoter at days 6 post-induction of differentiation are presented as mean ± standard error from two independent replicate experiments.

The ability of COS to inhibit the de-methylation of CpG_52, CpG_62, CpG_95 of the *LEP* promoter on days 2, 4 and 6PID was evaluated. COS had no effect on the de-methylation of any of these three CpG sites on days 2 and 4PID. However, on day 6PID, COS inhibited the de-methylation of CpG_52 (Figure 4A), CpG_62 (Figure 4B) and CpG_95 (Figure 4C).

### Comparison of the Expression of a Non-methylated and a Methylated LEP Promoter Construct

**Figure 5:**
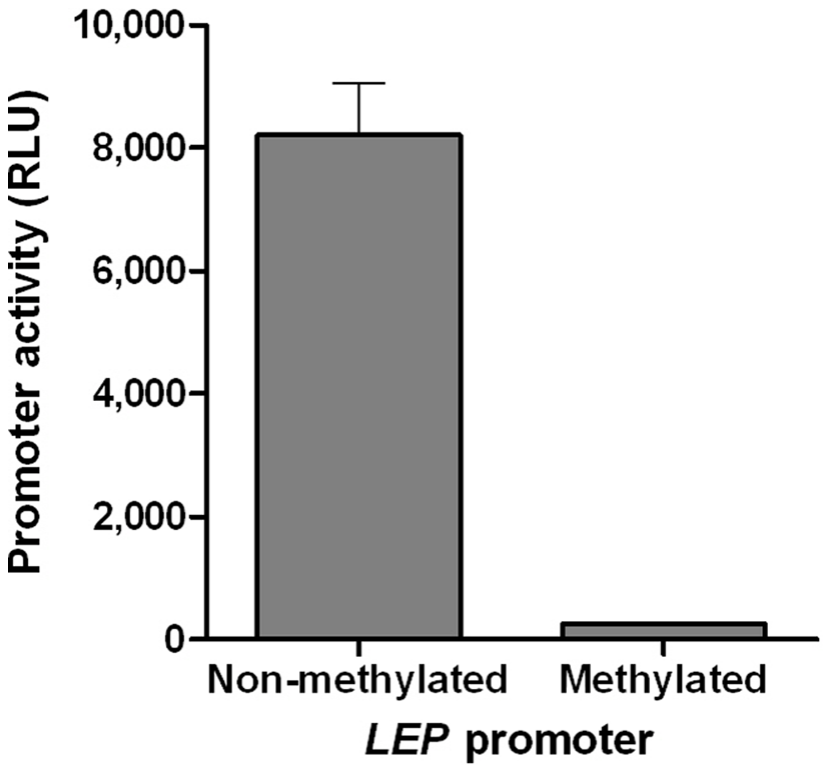
Transcriptional activities of an un-methylated and chemically methylated human *LEP* promoter in mouse 3T3-L1 pre-adipocytes.

To investigate whether the methylation of the *LEP* promoter is an underlying cause behind the absence of expression of leptin in pre-adipocytes (day 0PID), the expression of non-methylated and an *in-vitro* methylated *LEP* promoter constructs was evaluated in 3T3-L1 pre-adipocytes. The relative promoter transcriptional activity data (Figure 5) revealed that the non-methylated *LEP* promoter had a transcriptional activity of 8210.0±1462.0 RLU, while the *in-vitro* methylated *LEP* promoter had only limited transcriptional activity (247.5±21.7 RLU) in the pre-adipocytes.

## Discussion

We have previously determined that chitooligosaccharide (COS) suppresses the expression of a number of lipid metabolic pathway regulatory genes, including the *LEP* gene, and inhibits the accumulation of lipid in differentiating adipocytes [19]. Epigenetic regulation of the *LEP* gene was investigated in this study as an underlying mechanism of COS mediated inhibition of adipogenesis. Our results suggest that pre-adipocytes do not express leptin due to the presence of a highly methylated *LEP* promoter at that stage of cellular differentiation. The de-methylation of the *LEP* promoter occurs during the differentiation of pre-adipocytes to mature adipocytes, resulting in the expression of the *LEP* gene and subsequent production of leptin by the differentiated adipocytes. The process of de-methylation of selective CpG sites of the *LEP* promoter can be blocked by COS. This novel finding of inhibition of *LEP* gene expression by COS at an epigenetic level in adipocytes can be explored for manipulation of leptin gene expression, adipogenesis, energy homeostasis and obesity.

In this study, COS inhibited the de-methylation of three CpG sites (at −52, −62 and −95) in the *LEP* promoter. Of these three CpG sites, the most conspicuous de-methylation event was evident at CpG_52, which is present in a binding motif for the adipogenic transcription factor C/EBPα in the proximal promoter region of the *LEP* gene. This proximal promoter region (−55 to −47 bp), containing the C/EBPα site, is required for the trans-activation of *LEP* gene transcription [24] and subsequently leptin secretion [25], [26]. The fact that de-methylation of CpG_52 occurs during adipogenesis and de-methylated CpG_52 is required for binding of C/EBPα transcription factor and subsequent activation of the *LEP* gene expression [24], blocking of de-methylation of CpG_52 by COS most likely inhibited leptin expression. This might be an important mechanism by which COS can block the binding of C/EBPα, a key adipogenic transcription factor, and thus inhibits *LEP* gene expression in differentiating pre-adipocytes.

The mouse 3T3-L1 pre-adipocytes lack expression of *LEP* mRNA and protein, which indicated that the *LEP* gene as such is not expressed in the pre-adipocyte. However, as the differentiation of pre-adipocyte to mature adipocyte progresses, the abundance of *LEP* mRNA and protein increases. This is in agreement with previous reports on mouse 3T3-L1 adipocytes [17], [27] and human pre-adipocytes [16] which suggested that leptin is not produced by pre-adipocytes. We have compared the expression of an un-methylated and a chemically methylated *LEP* promoter in the pre-adipocyte and demonstrated that the presence of a highly methylated *LEP* promoter contributed to the lack of expression of leptin in pre-adipocytes.

In this experiment, a reduction of 4–12% methylation was recorded between the cells treated with the carrier only (COS 0 µg/ml) and highest concentration of COS (4800 µg/ml). These small but significant differences in the de-methylation could be due to: a) lack of complete differentiation of the cells at harvest (day 6PID), b) failure of every cell coming in contact to COS homogenously [28] and c) an inherent PCR bias associated with bisulfite PCR amplification of the genomic DNA [29]. Never-the-less methylation differences as little as 7% was previously reported to cause significant difference in gene expression [30]. Therefore, a reduction of 4–12% methylation recorded in this study due to the action of COS in three CpG sites is considered adequate for alteration in *LEP* gene expression.

COS being an anti-adipogenic bioactive compound, inhibits leptin expression at mRNA and protein levels in the differentiating pre-adipocyte. This inhibition of leptin secretion by adipocytes may alter the cellular fat reserve, which potentially has implication for conditions within the metabolic syndrome spectrum including obesity and type-2-diabetes. In both healthy and lean individuals, leptin works as a satiety signal reflecting the body energy reserve [31], [32]. In contrast, obese individuals generally have a high level of circulating leptin termed as ‘leptin resistance’ in which an excessive circulating leptin fails to signal for satiety and body energy reserve [32], [33]. Leptin interferes with glucose homeostasis and is reported to inhibit insulin gene expression and insulin secretion by pancreatic β-cells [33], [34], [35]. Therefore, COS mediated inhibition of leptin secretion may lead to an anti-diabetic effect in type-2-diabetes which is associated with secretion of excessive leptin leading to hypoinsulamia. Recent reports on anti-diabetic effect of chitosan in mice suggested that feeding of COS lead to an increase in glucose inducible insulin expression [36] and glucose uptake in streptozotocin induced diabetic mice [37].

The in-vitro data presented in this manuscript were obtained by using a homogeneous pre-adipocyte cell population and it was evident that COS can alter epigenetic modification of *LEP* gene expression during chemically induced adipogenesis. However, adipocyte development and differentiation in live animal is a complex and often an unsynchronized process where different populations of pre-adipocyte differentiate at various stages of development [38]. Therefore, the regulation of leptin promoter de-methylation by COS may be a complex phenomenon in the live animal which warrants further research.

Leptin also induces a pro-inflammatory response [31], [39], [40]. It induces the secretion of pro-inflammatory cytokines TNFα, IL-6 and IL-12 in macrophages [41] and causes a Th1 pro-inflammatory cytokine response [42]. If these *in-vitro* results translate *in-vivo*, COS mediated inhibition of leptin expression by adipocytes may be potentially helpful in reducing the chronic pro-inflammatory response in obese individuals. Interestingly, a number of anti-diabetic compounds such as thioglitazone [43], pioglitazone [44], rosiglitazone [45] and metformin [46] used to treat type-2-diabetes work through inhibition of leptin expression.

In conclusion, we have demonstrated that pre-adipocytes do not express the *LEP* gene due to the presence of a highly methylated *LEP* promoter. However, de-methylation of selective CpG sites of the *LEP* promoter, which occur during the differentiation process, can be inhibited by treatment with COS. This epigenetic inhibition of the *LEP* gene by COS in differentiating adipocytes could have implications for leptin regulation and for associated metabolic syndrome diseases such as obesity and type-2-diabetes.
